# Preoperative exercise capacity as a predictor of postoperative pulmonary complications in patients with esophageal cancer: a multicenter observational cohort study

**DOI:** 10.1007/s10388-026-01226-y

**Published:** 2026-06-30

**Authors:** Tomohiro Ikeda, Kazuhiro Noma, Kazuki Okura, Hiroki Sato, Sho Katayama, Yusuke Takahashi, Naoaki Maeda, Shunsuke Tanabe, Yusuke Sato, Yoshinori Fujiwara, Masanori Hamada, Toshifumi Ozaki, Tomohiko Nishigami, Toshiyoshi Fujiwara

**Affiliations:** 1 Department of Rehabilitation Medicine, Okayama University Hospital, 2-5-1 Shikatacho, Kita-ku, Okayama, 700-8558 Japan; 2 Graduate School of Comprehensive Scientific Research, Prefectural University of Hiroshima, Gakuencho 1-1, Mihara, Hiroshima 723-0053 Japan; 3 Department of Gastroenterological Surgery, Graduate School of Medicine, Dentistry and Pharmaceutical Sciences, Okayama University, 2-5-1 Shikatacho, Kita-ku, Okayama, 700-8558 Japan; 4 Center for Esophageal Disease, Okayama University Hospital, 2-5-1 Shikatacho, Kita-ku, Okayama, 700-8558 Japan; 5 Department of Rehabilitation Medicine, Akita University Hospital, 44-2 Hasunuma, Hiroomote, Akita, 010-8543 Japan; 6 Department of Physical Therapy, Faculty of Rehabilitation, Kawasaki University of Medical Welfare, 288 Matsushima, Kurashiki, Okayama 701-0193 Japan; 7 Department of Esophageal Surgery, Akita University Hospital, 44-2 Hasunuma, Hiroomote, Akita, 010-8543 Japan; 8 Department of Digestive Surgery, Kawasaki Medical School, 577 Matsushima, Kurashiki, Okayama 701-0192 Japan; 9 Department of Physical Therapy, Faculty of Health and Welfare, Prefectural University of Hiroshima, Gakuencho 1-1, Mihara, Hiroshima 723-0053 Japan

**Keywords:** Preoperative exercise, Pneumonia, Esophageal neoplasms, Postoperative complications, Logistic models

## Abstract

**Background:**

Postoperative pulmonary complications (PPCs) after esophageal cancer surgery remain a major concern. Preoperative exercise capacity has emerged as an important predictor. This study examined the clinical use of practical predictors—the 30-s chair stand test (CS-30) and the 6-min walk test (6MWT).

**Methods:**

We retrospectively analyzed patients with esophageal cancer from three institutions who underwent preoperative physical therapy between July 2021 and June 2023. Data completeness for the CS-30 and 6MWT was compared using McNemar’s test. Three logistic regression models predicting PPCs were developed: Model 1 (established risk factors), Model 2 (risk factors plus CS-30), and Model 3 (risk factors plus 6MWT). PPCs were defined as pneumonia or sputum retention requiring intervention (Clavien–Dindo grade ≥ 2). Model performance was evaluated using the area under the curve (AUC), and incremental predictive value was assessed using net reclassification improvement (NRI) and integrated discrimination improvement (IDI).

**Results:**

Among 213 patients (median age 68 years; 83% male), PPCs occurred in 57 (26.8%). Data completeness was significantly higher for the CS-30 (195/213, 91.5%) than the 6MWT (178/213, 83.6%; *p* < 0.001). The AUCs (95% confidence interval) were 0.775 (0.708–0.842) for Model 1, 0.786 (0.716–0.857) for Model 2, and 0.779 (0.712–0.846) for Model 3. NRI and IDI analyses showed that adding the CS-30 significantly improved reclassification and discrimination abilities.

**Conclusions:**

The CS-30 was more feasible than the 6MWT and significantly improved PPCs prediction. A perioperative management model for high-risk patients based on the CS-30 is recommended.

**Supplementary Information:**

The online version contains supplementary material available at https://doi.org/10.1007/s10388-026-01226-y.

## Introduction

Esophageal cancer is a prevalent malignancy worldwide [1]. Surgical resection remains the cornerstone of curative treatment; however, although advances in surgical and perioperative practices have successfully decreased mortality rates, complications remain high [2]. Postoperative pulmonary complications (PPCs) are among the most frequently observed adverse events following esophagectomy and are associated with increased mortality and long-term decline in health-related quality of life [3–5]. Therefore, early identification of high-risk patients with PPCs is essential for allocating limited medical resources and improving clinical outcomes.

Multiple clinical risk factors for PPCs have been reported, including advanced age, smoking history, impaired pulmonary function, comorbidities, and clinical stage in patients with esophageal cancer [6–9]. However, even with the current application of evidence-based perioperative strategies, PPCs continue to occur at a substantial rate [2]. In recent years, preoperative exercise capacity has garnered attention as a modifiable predictor of PPCs [10, 11]. The gold standard evaluation method, cardiopulmonary exercise testing (CPET), poses practical limitations in routine clinical settings due to the need for specialized equipment, trained personnel, and large testing areas [12]. This highlights the need for a simpler and more accessible method to evaluate exercise capacity in preoperative patients.

The 6-min walk test (6MWT) is a clinically practical method for assessing exercise capacity [13] and has been proposed as a potential predictor of postoperative outcomes [10]. However, it requires a 30-m walking path, appropriate footwear, and carries a potential risk of falls, and it cannot be performed while the patient is receiving intravenous infusions. These limitations may reduce its feasibility in certain perioperative settings, consistent with a report showing that the 6MWT could only be completed in 75.7% of patients with cancer awaiting major surgery [14]. High data completeness, an essential indicator of real-world feasibility, is critical in clinical practice to ensure the accurate prediction of PPCs. The 30-s chair-stand test (CS-30) is a simple physical function test performed using only a stopwatch and a chair [15] and has been used as an alternative assessment method. Previous studies have reported its validity as an exercise capacity index in both basic and clinical research [16–18]. Given its simplicity, the CS-30 may represent a more practical option for predicting PPCs in preoperative patients with esophageal cancer. However, to our knowledge, no study has evaluated its feasibility and predictive value in this population. Because single-institution analyses are often limited by small sample sizes and institutional bias, multicenter validation is essential to confirm the robustness and external generalizability of predictive models across diverse clinical settings.

The present multicenter retrospective study aimed to address this gap by examining (1) the feasibility of the CS-30 and 6MWT, based on data completeness, and (2) their added predictive value when incorporated into an existing PPC prediction model based on established clinical risk factors.

## Methods

### Design and participants

This was a multicenter, retrospective cohort study. All participants underwent curative esophagectomy, including thoracic and abdominal surgeries, at Okayama University Hospital, Akita University Hospital, and Kawasaki University Hospital in Japan between July 2021 and June 2023. Only patients who underwent preoperative physical function assessments were included. Exclusion criteria were as follows: patients with cerebrovascular or neuromuscular diseases expected to affect exercise capacity; patients requiring assistance with basic daily activities preoperatively; patients diagnosed with dementia preoperatively; patients who underwent two-stage esophagectomy; patients deemed inoperable during surgery and therefore underwent incomplete resection or had their surgery discontinued; and other patients deemed unsuitable by the physician. Given the retrospective design of this study, informed consent was obtained through an opt-out process. This study was approved by the Research Ethics Committee of Okayama University Hospital through a comprehensive review (2410-007) and was performed in accordance with the Declaration of Helsinki.

### Common perioperative physical therapy protocol in three hospitals

Physical therapists provided preoperative physical therapy five times per week as part of standardized perioperative management. It comprised supervised aerobic exercise, resistance training, respiratory exercises, and education. Postoperative physical therapy was provided five times per week and comprised progressive ambulation, respiratory physical therapy, and exercise therapy. On days without scheduled physical therapy sessions, progressive ambulation was encouraged and assisted by nursing staff. Details on these physical therapies are provided in Online Resource 1 (eTable 1).

### Data collection

The primary outcome was PPCs, defined as pneumonia or sputum retention disorders classified as Clavien–Dindo grade ≥ 2 occurring within 7 days after surgery [4]. The CS-30 and 6MWT data were collected as exercise capacity indices measured according to standardized clinical procedures. Preoperative exercise capacity assessments were performed by physical therapists with appropriate safety measures to prevent falls. The CS-30 was performed as previously described [15], using a 43-cm-high chair, with arms crossed over the chest, and counting the number of full stands in 30 s after one practice trial. The 6MWT followed American College of Chest Physicians guidelines on a 30-m flat course, with standardized verbal encouragement provided every minute [13]. Both CS-30 and 6MWT were assessed by a physical therapist within one week prior to surgery. When intravenous fluids or enteral nutrition were being administered, these were temporarily discontinued to perform the CS-30 or 6MWT. Clinical outcome indicators, including postoperative walking start date, length of hospital stays, and post-discharge outcomes (discharge/transfer/death), were used. Other variables collected included age, sex, clinical stage according to the UICC-TNM classification (8th edition) [19], tumor location, Charlson Comorbidity Index (CCI), malnutrition diagnosis according to the Global Leadership Initiative on Malnutrition criteria [20], forced vital capacity/forced expiratory volume in one second (FVC/FEV1), neoadjuvant therapy, surgical procedure, operating time, blood loss, and recurrent laryngeal nerve palsy.

### Statistical analysis

All statistical analyses were performed using R version 4.5.0 (R Foundation for Statistical Computing, Vienna, Austria). The R packages and code used for the analyses are listed in Online Resource 2. (1) To assess feasibility, we calculated the proportion of missing data for the CS-30 and 6MWT and descriptively compared data completeness between the two tests. McNemar’s test was used to statistically compare the proportions of available data. (2) To evaluate the incremental predictive value of the CS-30 and 6MWT for PPCs, three logistic regression models were constructed: Model 1 included established risk factors (age [6], sex [7], CCI ≥ 2 [8, 21], clinical stage ≥ II [9], malnutrition [7], and FEV1/FVC [7]); Model 2 added the CS-30 to Model 1; and Model 3 added the 6MWT to Model 1. The number of variables in each model was determined to maintain an events-per-variable ratio of ≥ 5 [22]. Model performance was compared using the area under the receiver operating characteristic curve (AUC) across the three models. Category-free net reclassification improvement (NRI), integrated discrimination improvement (IDI), and the likelihood ratio test were calculated to determine whether adding the CS-30 or 6MWT improved risk stratification beyond established predictors [23, 24]. Missing values in the regression models were handled using the random forest method [25]. Sensitivity analyses were conducted to confirm the robustness of the findings. First, the outcome variable was restricted to postoperative pneumonia alone, and the same three models were analyzed. Second, complete-case analyses were performed without imputation using the same modeling strategy to evaluate whether the results were consistent.

As an exploratory analysis, a receiver operating characteristic (ROC) curve analysis with PPCs as the outcome was employed to derive optimal cutoff values for CS-30 and 6MWT and to evaluate discriminative performance using the AUC. Missing data were imputed using a multiple imputation approach. Optimal thresholds were determined using the Youden index. Patients were then classified based on the calculated cutoff values, and postoperative outcomes were compared between groups. Appropriate statistical tests, the Wilcoxon signed-rank test, t test, chi-square test, or Fisher’s exact test, were used for comparisons. To further explore potential mechanisms linking exercise capacity to PPCs, additional models incorporating other physical function indicators, including handgrip strength (HGS) and gait speed, were constructed and evaluated using the same analytical procedures as those used for the exercise capacity. Finally, a descriptive analysis was conducted to characterize patients with missing CS-30 or 6MWT data.

## Results

### Preoperative clinicopathological characteristics and data completeness

**Fig. 1:**
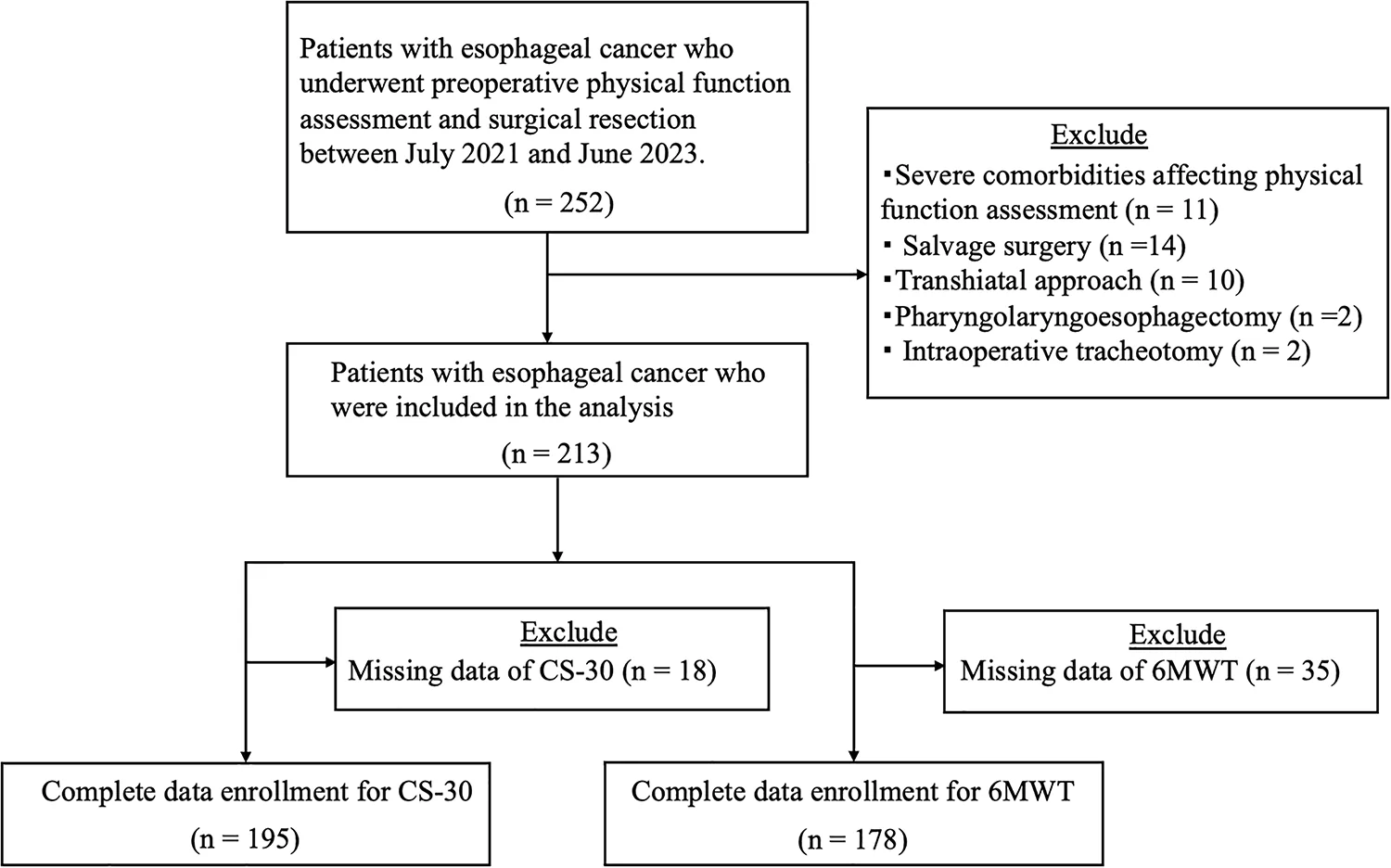
Flow diagram. *CS-30* 30-s chair-stand test, *6MWT* 6-min walk test

Of the 213 patients included, 148 were from Okayama University Hospital, 49 from Akita University Hospital, and 16 from Kawasaki University Hospital. The median [interquartile range, IQR] age was 68 [62–74] years, and 83% were male. PPCs occurred in 57 patients (26.8%), including pneumonia in 42 patients (19.7%). The median [IQR] CS-30 was 16 [13–21] 13–21 repetitions, and the median 6MWT was 480 [420–545] m (Table 1). Complete CS-30 data were available for 195 patients (91.5%), and complete 6MWT data were available for 178 patients (83.6%) (Fig. 1). Among the two assessments, 3 patients (1.4%) lacked only CS-30 data, 20 patients (9.4%) lacked only 6MWT data, and 15 patients (7.0%) lacked both. Data completeness was significantly higher for the CS-30 than for the 6MWT, according to McNemar’s test (p < 0.001).

**Table 1:** Preoperative clinicopathological characteristics

	Overall, N = 213^a^
Age (y)	69 (62, 74)
Sex (male)	172 (81%)
BMI (kg/m^2^)	21.6 (19.6, 23.4)
*Location*	
Ce	8 (4%)
Te	180 (84%)
Ge	25 (12%)
Histology	
SCC	189 (89%)
AC	18 (9%)
Other	6 (3%)
Brinkman index(≥ 800)	74 (35%)
CCI (≥ 2)	57 (27%)
Clinical Stage (≥ III)	103 (48%)
T (≥ 3)	113 (53%)
N (Positive)	118 (55%)
M (Positive)	1 (1%)
*Neoadjuvant therapy*	
None	72 (34%)
NACT	119 (56%)
NACRT	22 (10%)
Hemoglobin (mg/dl)	11.8 (10.5, 13.2)
Albumin (g/dl)	3.8 (3.5, 4.1)
Malnutrition	59 (28%)
CS-30 (repetition), NA = 18	16 (13, 21)
6MWT (m), NA = 35	480 (420, 540)
HGS (kg), NA = 6	34 (27, 38)
Gait speed (m/s), NA = 8	1.28 (1.12, 1.39)
FEV1/FVC (%)	86 (76, 100)

*BMI* body mass index, *Ae* abdominal esophagus, *Te* thoracic esophagus, *Ce* cervical esophagus, *SCC* squamous cell carcinoma, *AC* adenocarcinoma, *Brinkman index* multiplying the average number of cigarettes smoked per day by the number of years the person has smoke, *CCI* Charlson comorbidity index, *NACT* Neoadjuvant chemotherapy, *NACRT* Neoadjuvant chemoradiotherapy, *Malnutrition* diagnosis by the Global Leadership Initiative on Malnutrition criteria, *CS-30* 30-s chair-stand test, *6MWT* 6-min walk test, *HGS* hand grip strength *FEV1/FVC* forced expiratory volume in 1 s / forced vital capacity

^a^Median (IQR); n (%)

### Prediction models for PPCs

**Fig. 2:**
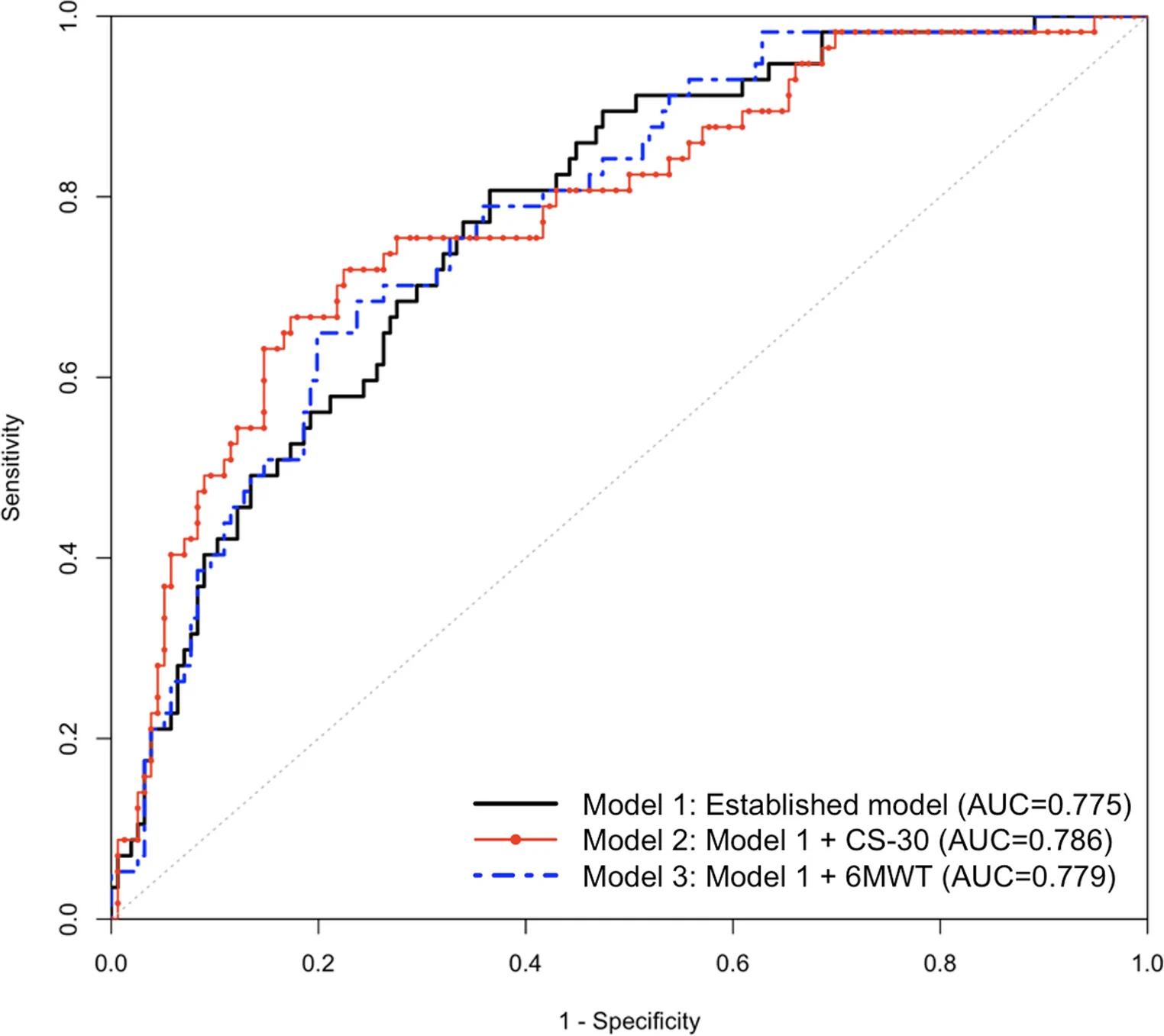
Receiver operating characteristic curve for evaluating the PPCs prediction model, including the CS-30 and 6MWT (random forest imputation analysis). *PPCs* Postoperative pulmonary complications = pneumonia or sputum excretion difficulty, *Established model* model including established risk factors for PPCs = age, sex, Charlson Comorbidity Index ≥2, clinical stage ≥II, malnutrition, and forced vital capacity/forced expiratory volume in one second, *CS-30* 30-s chair-stand test, *6MWT* 6-min walk test

The main analysis was modeled using data from 213 patients, with missing values handled using imputation. The AUCs (95% confidence interval [95% CI]) were 0.775 (0.708–0.842) for Model 1, 0.786 (0.716–0.857) for Model 2, and 0.779 (0.712–0.846) for Model 3 (Fig. 2). When added to the established model, the CS-30 substantially improved the discrimination ability, as demonstrated by a significant category-free NRI of 0.427 (95% CI, 0.134–0.719; *p* = 0.004) and an IDI 0.048 (0.018–0.076; *p* = 0.001). In contrast, adding the 6MWT did not significantly improve reclassification or discrimination (Table 2). The likelihood ratio test indicated that adding CS-30 significantly improved Model 1 (p = 0.007), whereas adding the 6MWT did not provide a significant improvement (p = 0.540). Sensitivity analyses using postoperative pneumonia as the outcome demonstrated similar results (Online Resource 1: eTable 2; Online Resource 3: eFig. 1). In the complete-case analysis, adding the CS-30 showed a trend toward improved NRI (0.327 [− 0.043, 0.671]; *p* = 0.056) (Online Resource 1: eTable 3; Online Resource 3: eFig. 2) .

**Table 2:** NRI and IDI for discrimination ability of CS-30 and other physical function indicators in combination with the known risk factors for PPCs (random forest imputation analysis)

	Category-free NRI		IDI	
	NRI value (95% CI)	*p*-value	IDI value	*p*-value
*Established model*				
+ CS-30	0.427 (0.134, 0.719)	0.004*	0.048 (0.018, 0.076)	0.001*
+ 6MWT	0.267 (-0.034, 0.568)	0.082	0.007 (-0.004, 0.019)	0.217
+ HGS	0.033 (-0.039, 0.103)	0.379	0.001 (-0.002, 0.002)	0.950
+ Gait speed	0.318 (-0.021, 0.615)	0.036*	0.004 (-0.006, 0.014)	0.413

*NRI* net reclassification improvement, *IDI* integrated discrimination improvement, *CS-30* 30-s chair stand test, *PPCs* Postoperative pulmonary complications = pneumonia or sputum excretion difficulty, *Established model* model including established risk factors for PPCs = age, sex, Charlson Comorbidity Index ≥2, clinical stage ≥II, malnutrition, and forced vital capacity/forced expiratory volume in one second, *6MWT* 6-min walk test, *HGS* handgrip strength

^*^Statistically significant difference (*p* < 0.05)

**Fig. 3:**
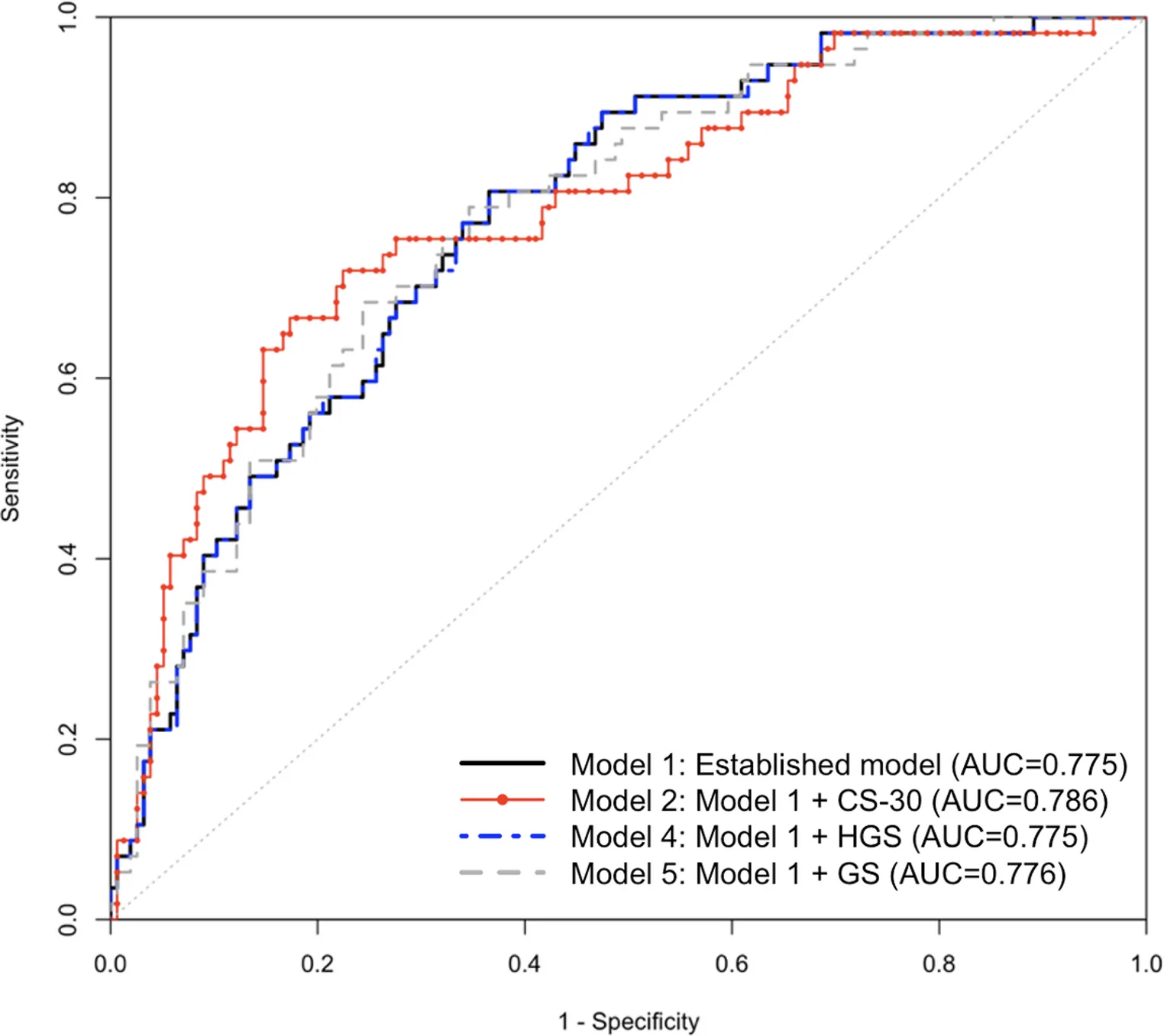
ROC curve for evaluating the PPCs prediction model, including the CS-30 and other physical function indicators (random forest imputation analysis). *ROC* receiver operating characteristic, *PPCs* Postoperative pulmonary complications = pneumonia or sputum excretion difficulty, *Established model* model including established risk factors for PPCs = age, sex, Charlson Comorbidity Index ≥2, clinical stage ≥II, malnutrition, and forced vital capacity/forced expiratory volume in one second, *CS-30* 30-s chair-stand test*, HGS* handgrip strength, *GS* gait speed

### Exploratory analysis

ROC analysis identified an optimal CS-30 cutoff of 15 repetitions for predicting PPCs, with an AUC of 0.655 (0.568, 0.741), sensitivity of 0.561, and specificity of 0.726. The optimal 6MWT cutoff was 510 m for predicting PPCs with an AUC of 0.635 (0.557, 0.713), sensitivity of 0.858, and specificity of 0.429. Among patients with CS-30 scores below the cutoff value, postoperative ambulation onset was delayed (3 vs. 2 days), length of stay (LOS) was prolonged (27 vs. 22 days), and the discharge rate was lower (73% vs. 86%) (Table 3). Among patients with 6MWT distances below the cutoff value, postoperative ambulation onset was delayed (3 vs. 2 days), LOS was prolonged (26 vs. 20 days), and the discharge rate was lower (75% vs. 97%) (Table 4). After adding HGS and gait speed to Model 1, the AUCs (95% CIs) were 0.775 (0.709–0.843) and 0.776 (0.711–0.844), respectively (Fig. 3). HGS and gait speed did not significantly improve reclassification and discrimination abilities (Table 2). A descriptive analysis of clinical characteristics revealed that patients with missing 6MWT data were generally older, had lower levels of physical function, and experienced poorer clinical outcomes (Online Resource 1: eTable 5).

**Table 3:** Comparison of surgical and postoperative information according to CS-30 scores (< 15 vs. ≥ 15 repetition)

Characteristics	Overall, N = 213^a^	CS-30 < 15 repetition, N = 64^a^	CS-30 ≥ 15 repetition, N = 131^a^	*p*-value^b^
*Surgical information*				
LND (3 area)	126 (59%)	44 (69%)	75 (57%)	0.124
Robot	101 (47%)	30 (47%)	65 (50%)	0.719
Thoracoscopic	106 (50%)	34 (53%)	60 (46%)	0.337
Thoracotomy	7 (3%)	0 (0%)	7 (5%)	0.098
Laparoscopic	141 (66%)	47 (73%)	79 (60%)	0.072
Laparotomy	71 (33%)	17 (27%)	51 (39%)	0.089
Time (min)	638 (552, 717)	658 (542, 721)	640 (565, 716)	0.932
Bleeding (ml)	143 (70, 250)	130 (60, 195)	150 (70, 310)	0.142
*Postoperative information*				
First gait (days)	3 (2, 4)	3 (3, 6)	2 (2, 3)	< 0.001*
LOS (days)	23 (19, 31)	27 (20, 35)	22 (18, 27)	0.006*
Goal				0.040*
Discharge	169 (79%)	47 (73%)	113 (86%)	
Hospital transfer	43 (20%)	17 (27%)	17 (13%)	
Death	1 (1%)	0 (0%)	1 (1%)	
*Complications*				
Pneumonia	42 (20%)	19 (30%)	19 (15%)	0.012*
Pneumonia/Sputum excretion difficulty	57 (27%)	27 (42%)	26 (20%)	< 0.001*
Anastomotic Leakage	10 (5%)	2 (3%)	7 (5%)	0.720
RLNP	30 (14%)	10 (16%)	19 (15%)	0.836
SSI	9 (4%)	1 (2%)	8 (5%)	0.276
Poaf	11 (5%)	3 (5%)	6 (5%)	> 0.009

18 patients had missing CS-30 and were excluded from this comparison

*CS-30* 30-s chair-stand test, *LND* lymph node density, *LOS* length of stay, *RLNP* recurrent laryngeal nerve palsy, *SSI* surgical site infection, *Poaf* postoperative atrial fibrillation

^*^Indicates statistically significant difference (*p* < 0.05)

^a^Median (IQR); n (%)

^b^Wilcoxon rank sum test; Pearson’s chi-squared test; Fisher’s exact test

**Table 4:** Comparison of surgical and postoperative information according to 6MWT scores (< 510 vs. ≥ 510 m)

Characteristics	Overall, N = 213^a^	6MWT < 510 m, N = 107^a^	6MWT ≥ 510 m, N = 71^a^	*p*-value^b^
*Surgical information*				
LND (3 area)	126 (59%)	67 (63%)	40 (56%)	0.245
Robot	101 (47%)	58 (54%)	31 (44%)	0.168
Thoracoscopic	106 (50%)	47 (44%)	36 (51%)	0.375
Thoracotomy	7 (3%)	3 (3%)	4 (6%)	0.439
Laparoscopic	141 (66%)	66 (62%)	47 (66%)	0.540
Laparotomy	71 (33%)	41 (38%)	23 (32%)	0.420
Time (min)	638 (552, 717)	652 (559, 721)	648 (565, 721)	0.950
Bleeding (ml)	143 (70, 250)	163 (75, 371)	127 (50, 210)	0.047
*Postoperative information*				
First gait (days)	3 (2, 4)	3 (2, 5)	2 (2, 3)	< 0.001*
LOS (days)	23 (19, 31)	26 (21, 34)	20 (17, 25)	< 0.001*
Goal				< 0.001*
Discharge	169 (79%)	80 (75%)	69 (97%)	
Hospital transfer	43 (20%)	26 (24%)	2 (3%)	
Death	1 (1%)	1 (1%)	0 (0%)	
*Complications*				
Pneumonia	42 (20%)	25 (23%)	7 (10%)	0.022*
Pneumonia/Sputum excretion difficulty	57 (27%)	37 (35%)	10 (14%)	0.002*
*Anastomotic Leakage*	10 (5%)	3 (3%)	6 (9%)	0.159
RLNP	30 (14%)	20 (19%)	7 (10%)	0.108
SSI	9 (4%)	4 (4%)	4 (6%)	0.715
Poaf	11 (5%)	6 (6%)	3 (4%)	> 0.999

35 patients had missing 6MWT and were excluded from this comparison

*6MWT* 6-min walk test*, LND* lymph node density, *LOS* length of stay, *PPCs* Postoperative pulmonary complications = pneumonia or sputum excretion difficulty, *RLNP* recurrent laryngeal nerve palsy, *SSI* surgical site infection, *Poaf* postoperative atrial fibrillation

*Indicates statistically significant difference (*p* < 0.05)

^a^Median (IQR); n (%)

^b^Wilcoxon rank sum test; Pearson’s chi-squared test; Fisher’s exact test

## Discussion

This multicenter retrospective study evaluated the data completeness of the preoperative CS-30 and 6MWT and compared their predictive performance for PPCs in patients undergoing esophagectomy. Two main findings emerged: (1) the CS-30 demonstrated higher data completeness than the 6MWT, and (2) incorporating the CS-30 in a model of established clinical risk factors significantly improved the prediction of PPCs, whereas adding the 6MWT provided no additional benefit.

To our knowledge, this is the first multicenter real-world study to evaluate both the data completeness and incremental predictive value of preoperative exercise capacity assessments, specifically the CS-30 and 6MWT, for PPCs in patients with esophageal cancer. Furthermore, the predictive capacity of the CS-30 extended beyond PPCs, as scores below an exploratory cutoff (< 15 repetitions) were associated with longer hospital stays and lower discharge rates. These findings suggest that the CS-30 is clinically feasible and effective for identifying patients experiencing difficulties in postoperative recovery.

In this multicenter cohort study, the data completeness rate was 91.5% for the CS-30 and 83.6% for the 6MWT. Notably, among the two assessments, isolated missing CS-30 data were observed in 1.4% of patients, whereas isolated missing 6MWT data occurred in 9.4%. These differences are likely attributable to the minimal requirements of the CS-30, which requires only a chair and a stopwatch and can be performed in a small clinical space. Similar high data completeness rates (> 99%) have been reported in older individuals with heart failure [26], whereas the 6MWT has shown lower feasibility (75.7–82.1%) in preoperative patients with cancer [10, 14]. These findings support the CS-30 as a practical and applicable tool for preoperative risk assessment in patients undergoing esophagectomy.

Another key finding was the significant incremental predictive value of the CS-30 for PPCs. When added to the model of established risk factors, the CS-30 increased the AUC from 0.775 to 0.786. Incremental predictive value analyses (NRI and IDI) further support the CS-30’s added predictive value, demonstrating both statistically and clinically relevant improvements in risk reclassification. This finding suggests that the CS-30 captures dimensions of patient vulnerability not fully reflected by traditional risk factors, such as age, comorbidities, or malnutrition, thereby contributing to the prediction of PPCs. Three mechanisms may explain the association between CS-30 performance and PPCs. First, the CS-30 mainly assesses exercise capacity [17, 18], which is a key component in predicting PPCs in patients with esophageal cancer [10]. In the exploratory analyses of this study, other indicators of muscle strength and mobility, such as HGS and gait speed, did not provide additional predictive value, highlighting the particular importance of exercise capacity for predicting PPCs. Second, the CS-30 may function as a more comprehensive indicator of systemic physical frailty [27], as related parameters, including grip strength [28], physical function [6], and respiratory muscle strength [29], are all independently associated with PPCs. Third, patients with poor CS-30 performance (< 15 repetitions) in our study experienced delayed postoperative ambulation, a recognized risk factor for PPCs [30]. These findings are further supported by recent real-world evidence from patients undergoing lung resection, in which the CS-30 also independently predicted postoperative complications [31], reinforcing its clinical relevance across thoracic surgical populations.

In contrast, the 6MWT did not demonstrate significant additive value for PPC prediction in this cohort. This may reflect its lower data completeness, which may have excluded high-risk patients unable to perform the test. This interpretation was supported by descriptive analyses of missing 6MWT data and clinical characteristics, which indicated that patients with missing data tended to be older, generally had reduced physical function, and experienced poorer clinical outcomes. These results emphasize the need to consider feasibility and predictive accuracy when selecting preoperative assessment tools. From a physiological perspective, while 6MWT primarily assesses exercise capacity, CS-30 reflects not only exercise capacity but also related domains, including standing ability, an established predictor of PPCs [6], and peak cough flow, which has been strongly associated with PPCs [32]. Collectively, these findings suggest that CS-30 captures physiological domains relevant to PPCs development more comprehensively than 6MWT, which may improve PPCs risk prediction.

These findings have several practical implications. Given its high feasibility, minimal resource requirements, and robust predictive performance, the CS-30 can be readily integrated into routine preoperative evaluations across diverse clinical environments. The multicenter design of this study enhances its generalizability, suggesting that the CS-30 is applicable not only in tertiary centers but also in community hospitals lacking CPET or adequate space for the 6MWT. Incorporating the CS-30 into clinical decision-making may facilitate earlier referral of high-risk patients to rehabilitation services and support more individualized perioperative rehabilitation strategies. In this context, multimodal prehabilitation approaches centered on supervised aerobic exercise [33], with an emphasis on improving exercise capacity as reflected by CS-30 performance, may be especially relevant for these patients. For patients undergoing neoadjuvant chemotherapy, initiating such interventions during chemotherapy rather than only in the immediate preoperative phase may be particularly beneficial [34, 35]. Furthermore, our data indicate that patients classified as high risk based on CS-30 experience delayed postoperative ambulation. Accordingly, identifying and addressing barriers to ambulation through a multidisciplinary approach and actively promoting early mobilization may also contribute to the prevention of PPCs. Additionally, the use of cutoff values is important in clinical practice; however, the optimal CS-30 cutoff value calculated from our cohort was 15, differing from the previously reported cutoff value of 13 for Australian patients prior to major cancer surgery. These differences may reflect variations in body stature, such as height and weight, between Asian and European populations. Therefore, while our findings support the robustness of CS-30 as a risk stratification tool, the optimal cutoff value requires validation in each region. Furthermore, consideration of gender-appropriate cutoff values is also required.

This study has several limitations. First, its retrospective design introduces potential selection and information biases. Second, the predictive accuracy of the models may be limited by the restricted scope of the incorporated risk factors for PPCs. Because the primary aim of this study was to identify preoperative predictors of PPCs for early risk stratification and support decision-making in perioperative management, including prehabilitation, key intraoperative variables, such as operative time, blood loss, and surgical approach, were intentionally excluded. Third, data for the predictive models were handled with imputation for missing values, which, although a valid statistical approach, is not a perfect substitute for complete data. Finally, the findings may not apply to patients with knee or back pain who are unable to perform the CS-30. Future prospective studies are warranted to validate our findings and establish a definitive optimal cutoff value for the CS-30 in this patient population. Furthermore, it is important to develop targeted prehabilitation strategies for high-risk patients identified through CS-30 scores, including interventions to improve exercise capacity, and to determine their effect on PPCs.

In conclusion, this multicenter study demonstrated that the CS-30 is more feasible than the 6MWT, with a higher data completion rate in preoperative patients with esophageal cancer. Moreover, incorporating the CS-30 into established PPC prediction models significantly improved predictive performance. Given its simplicity and practicality, integrating the CS-30 into routine preoperative assessments may enhance risk stratification and support individualized perioperative management.

## Supplementary Information

Below is the link to the electronic supplementary material.

Supplementary Material 1

Supplementary Material 2

Supplementary Material 3

## Data Availability

The data sets used in this study are available from the corresponding author upon reasonable request.
