# Supplementary material for: Preoperative exercise capacity as a predictor of postoperative pulmonary complications in patients with esophageal cancer: a multicenter observational cohort study
Source: Esophagus. 2026 Jun 30;23(4):791–800. doi: 10.1007/s10388-026-01226-y (PMC13723555; doi:10.1007/s10388-026-01226-y)
Supplement: Supplementary file 1 [file 10388_2026_1226_MOESM1_ESM.docx]

**Title: Preoperative exercise capacity as a predictor of postoperative respiratory complications in patients with esophageal cancer: a multicenter observational cohort study**

Journal name: Esohagus

Authors: Tomohiro Ikeda, MSc^1, 2^, Kazuhiro Noma, MD, PhD^3, 4^**^*^**, Kazuki Okura, PhD^5^, Hiroki Sato, PhD^6^, Sho Katayama^1^ ,Yusuke Takahashi, PhD^5^, Naoaki Maeda, MD, PhD^3, 4^, Shunsuke Tanabe, MD, PhD^3, 4^, Yusuke Sato, MD, PhD^7^, Yoshinori Fujiwara, MD, PhD^8^, Masanori Hamada, MD, PhD^1^, Toshifumi Ozaki, MD, PhD^1^, Tomohiko Nishigami, PhD^9^, Toshiyoshi Fujiwara, MD, PhD^3,4^

Affiliations: ^1^Department of Rehabilitation Medicine, Okayama University Hospital, 2-5-1 Shikatacho, Kita-ku, Okayama 700-8558, Japan

^2^Graduate School of Comprehensive Scientific Research, Prefectural University of Hiroshima, Gakuencho 1-1, Mihara, Hiroshima 723-0053, Japan

^3^Department of Gastroenterological Surgery, Graduate School of Medicine, Dentistry and Pharmaceutical Sciences, Okayama University, 2-5-1 Shikatacho, Kita-ku, Okayama 700-8558, Japan

^4^Center for Esophageal Disease, Okayama University Hospital, 2-5-1 Shikatacho, Kita-ku, Okayama 700-8558, Japan

^5^Department of Rehabilitation Medicine, Akita University Hospital, 44-2 Hasunuma, Hiroomote, Akita 010-8543, Japan

^6^Department of Physical Therapy, Faculty of Rehabilitation, Kawasaki University of Medical Welfare, Kurashiki, 288 Matsushima, Okayama 701-0193, Japan.

^7^Department of Esophageal Surgery, Akita University Hospital, 44-2 Hasunuma, Hiroomote, Akita 010-8543, Japan

^8^Department of Digestive Surgery, Kawasaki Medical School, Kurashiki, 577 Matsushima, Okayama 701-0192, Japan

^9^Department of Physical Therapy, Faculty of Health and Welfare, Prefectural University of Hiroshima, Gakuencho 1-1, Mihara, Hiroshima 723-0053, Japan

**^*^Corresponding author:** Kazuhiro Noma

Department of Gastroenterological Surgery, Graduate School of Medicine, Dentistry and Pharmaceutical Sciences, Okayama University, 2-5-1 Shikatacho, Kita-ku, Okayama 700-8558, Japan

E-mail address: [knoma@md.okayama-u.ac.jp](mailto:knoma@md.okayama-u.ac.jp)

Telephone number: +81-86-235-7752; Fax number: +81-86-235-7751

eTable 1. Common perioperative physical therapy protocol in three hospitals

| Phase | Type | Supervised | Frequency | Time | intensity | Note | |  |
| --- | --- | --- | --- | --- | --- | --- | --- | --- |
| Preoperative | Aerobic exercise | Supervised  +Unsupervised | 5 sessions/week | 10-20 min | Exercise intensity was progressively increased to achieve a target of 3–4 on the modified Borg scale (“somewhat hard”). | | Tailored to baseline physical function and cancer-related symptoms (such as fatigue and loss of appetite). | |
|  | Resistance training | Supervised  +Unsupervised | 5 sessions/week | 5-10 min | Exercise intensity was progressively increased to achieve a target of 5 on the modified Borg scale (“hard”). | |  | |
|  | Respiratory training | Supervised  +Unsupervised | 7 sessions/week | 5-10 min | Maximal deep breathing exercises were performed using respiratory training devices (e.g., incentive spirometers), with a target of two sets of 30 breaths. Instruct on diaphragmatic breathing techniques, aspiration techniques and thoracic stretching. Physical therapist regularly verifies that there are no errors in the training methods. | |  | |
|  | Education | Supervised | 5 sessions/week | 5-10 min | Explain how early ambulation after surgery contributes to postoperative outcomes. Explain avoidance movements related to postoperative pain. Patients were instructed to perform self-exercise combining aerobic exercise, resistance training, and respiratory training according to the supervised physical therapy program. | |  | |
| Postoperative | Early mobilization | Supervised | 5 sessions/week (+2 sessions/week with nurse) | 10-20 min | Postoperative day 1, patients underwent progressive mobilization (sitting, standing and walking) based on their vital signs, pain levels, and other physical symptoms. | | Tailored to baseline physical function and postoperative- symptoms (such as pain and respiratory or circulatory problems). | |
|  | Respiratory training | Supervised  +Unsupervised | 5 sessions/week | 5-10 min | Instruct on diaphragmatic breathing techniques, aspiration techniques and thoracic stretching. Physical therapist regularly verifies that there are no errors in the training methods. | |  | |
|  | Education | Supervised | 5 sessions/week | 5-10 min | Explain how early ambulation after surgery contributes to postoperative outcomes. Explain avoidance movements related to postoperative pain. For patients who achieve independent sitting, instruct them to gradually increase sitting frequency and duration. For patients who achieve independent walking, instruct them to gradually increase walking frequency and distance. | |  | |
| Postoperative (Achieved independent walking within the hospital) | Aerobic exercise | Supervised  +Unsupervised | 5 sessions/week | 10-20 min | Exercise intensity was progressively increased to achieve a target of 3–4 on the modified Borg scale (“somewhat hard”). | | Tailored to baseline physical function and postoperative- symptoms (such as malnutrition, pain, continuing cough and respiratory or circulatory problems). | |
|  | Resistance training | Supervised  +Unsupervised | 5 sessions/week | 5-10 min | Exercise intensity was progressively increased to achieve a target of 5 on the modified Borg scale (“hard”). | |  | |
|  | Respiratory training | Supervised  +Unsupervised | 7 sessions/week | 5-10min | Maximal deep breathing exercises were performed using respiratory training devices (e.g., incentive spirometers), with a target of two sets of 30 breaths. Instruct on diaphragmatic breathing techniques, aspiration techniques and thoracic stretching. Physical therapist regularly verifies that there are no errors in the training methods. | |  | |
|  | Education | Supervised | 5 sessions/week | 5-10min | Explain that postoperative exercise therapy is important for enhancing quality of life after discharge. Patients were instructed to perform self-exercise combining aerobic exercise, resistance training, and respiratory training according to the supervised physical therapy program. | |  | |

eTable 2. NRI and IDI for the discrimination ability of the CS-30 and other physical function indicators in combination with known risk factors for pneumonia (random forest imputation analysis).

|  | Category-free NRI |  | IDI |  |
| --- | --- | --- | --- | --- |
|  | NRI value (95% CI) | *p*-value | IDI value | *p*-value |
| Established model |  |  |  |  |
| + CS-30 | 0.334 (0.016, 0.644) | 0.042* | 0.038 (0.008, 0.068) | 0.013* |
| + 6MWT | 0.052 (-0.285, 0.389) | 0.763 | 0.001 (-0.004, 0.006) | 0.696 |
| + HGS | 0.137 (-0.198, 0.472) | 0.422 | 0.003 (-0.005, 0.010) | 0.500 |
| + Gait speed | 0.349 (0.020, 0.679) | 0.038* | 0.006 (-0.008, 0.019) | 0.429 |

* Statistically significant difference (*p* <0.05).

**NRI**, net reclassification improvement; **IDI**, integrated discrimination improvement

**CS-30,** 30-second chair stand test**; 6MWT,** 6-minute walk test; **HGS**, handgrip strength

eTable 3. NRI and IDI for the discrimination of CS-30 and other physical function indicators in combination with known risk factors for PPCs (complete case analysis)

|  | Category-free NRI |  | IDI |  |
| --- | --- | --- | --- | --- |
|  | NRI value (95% CI) | *p*-value | IDI value | *p*-value |
| Established model |  |  |  |  |
| + CS-30 | 0.327 (-0.043, 0.671) | 0.056 | 0.047 (0.015, 0.078) | 0.004* |
| + 6MWT | 0.105 (-0.236, 0.445) | 0.547 | 0.001 (-0.005, 0.008) | 0.683 |
| + HGS | 0.033 (-0.279, 0.345) | 0.835 | 0.000 (-0.002, 0.002) | 0.950 |
| + Gait speed | 0.295 (-0.010, 0.600) | 0.058 | 0.004 (-0.006, 0.013) | 0.458 |

* Statistically significant difference (*p* <0.05).

**NRI**, net reclassification improvement; **IDI**, integrated discrimination improvement

**CS-30,** 30-second chair stand test**; 6MWT,** 6-minute walk test; **HGS**, handgrip strength

eTable 4. Missing data in the CS-30 and clinical characteristics

| Characteristic | Overall,  N = 213^a^ | CS-30 complete,  N = 195^a^ | CS-30 missing,  N = 18^a^ | *p*-value^b^ |
| --- | --- | --- | --- | --- |
| Preoperative information |  |  |  |  |
| Age | 68 (10) | 68 (9) | 71 (12) | 0.066 |
| Sex (male) | 172 (81%) | 161 (83%) | 11 (61%) | 0.053 |
| BMI | 21.8 (3.2) | 21.8 (3.2) | 21.3 (3.0) | 0.521 |
| Brinkman index (≥800) | 74 (35%) | 65 (33%) | 9 (50%) | 0.155 |
| CCI (≥2) | 57 (27%) | 50 (26%) | 7 (39%) | 0.266 |
| Clinical Stage (≥III) | 151 (71%) | 142 (73%) | 9 (50%) | 0.041* |
| Malnutrition | 59 (28%) | 54 (28%) | 5 (28%) | >0.999 |
| FEV1/FVC (%) | 75 (10) | 75 (10) | 75 (10) | 0.886 |
| CS-30 (repetition) | 17.1 (5.6) | 17.1 (5.6) | NA (NA, NA) |  |
| 6MWT (m) | 474 (101) | 475 (101) | 412 (96) | 0.371 |
| Gait speed (m/s) | 1.27 (0.24) | 1.28 (0.23) | 1.00 (0.23) | 0.001* |
| HGS (kg) | 33 (9) | 33 (9) | 29 (11) | 0.224 |
| Postoperative information |  |  |  |  |
| PPCs | 57 (27%) | 50 (26%) | 7 (39%) | 0.266 |
| Pneumonia | 42 (20%) | 36 (18%) | 6 (33%) | 0.132 |
| LOS (days) | 23 (19, 31) | 23 (19, 31) | 30 (22, 42) | 0.050 |
| Discharge | 44 (21%) | 35 (18%) | 9 (50%) | 0.004* |
| ^a^Mean (SD); Median (IQR); n (%) | | | | |
| ^b^Wilcoxon rank sum test; t test; Pearson's chi-squared test; Fisher's exact test  *Indicates statistically significant difference (*p* <0.05)  **BMI**, body mass index; **CCI,** Charlson comorbidity index; **Malnutrition**, diagnosis by the Global Leadership Initiative on Malnutrition criteria; **FEV1/FVC,** forced expiratory volume in 1 second / forced vital capacity; **CS-30,** 30-second chair-stand test**; 6MWT,** 6-minute walk test**; HGS**, hand grip strength, **PPCs**, Postoperative pulmonary complications = pneumonia or sputum excretion difficulty; **LOS**, length of stay | | | | |

eTable 5. Missing data in the 6MWT and clinical characteristics

| Characteristic | Overall,  N = 213^a^ | 6MWT complete,  N = 178^a^ | 6MWT missing,  N = 35^a^ | *p*-value^b^ |
| --- | --- | --- | --- | --- |
| Preoperative information |  |  |  |  |
| Age | 68 (10) | 67 (9) | 70 (11) | 0.165 |
| Sex (male) | 172 (81%) | 149 (84%) | 23 (66%) | 0.014* |
| BMI | 21.8 (3.2) | 21.9 (3.3) | 21.2 (2.8) | 0.223 |
| Brinkman index (≥800) | 74 (35%) | 58 (33%) | 16 (46%) | 0.136 |
| CCI (≥2) | 57 (27%) | 47 (26%) | 10 (29%) | 0.791 |
| Clinical Stage (≥III) | 151 (71%) | 132 (74%) | 19 (54%) | 0.018* |
| Malnutrition | 59 (28%) | 48 (27%) | 11 (31%) | 0.590 |
| FEV1/FVC (%) | 75 (10) | 76 (9) | 72 (11) | 0.075 |
| CS-30 (repetition) | 17.1 (5.6) | 17.4 (5.6) | 14.0 (4.6) | 0.005* |
| 6MWT (m) | 480 (420, 541) | 480 (420, 541) | NA (NA, NA) |  |
| Gait speed (m/s) | 1.27 (0.24) | 1.29 (0.23) | 1.10 (0.26) | <0.001* |
| HGS (kg) | 33 (9) | 34 (9) | 29 (9) | 0.017* |
| Postoperative information |  |  |  |  |
| PPCs | 57 (27%) | 44 (25%) | 13 (37%) | 0.129 |
| Pneumonia | 42 (20%) | 30 (17%) | 12 (34%) | 0.018* |
| LOS (days) | 23 (19, 31) | 23 (19, 31) | 27 (22, 33) | 0.114 |
| Discharge | 44 (21%) | 29 (16%) | 15 (43%) | <0.001* |
| ^a^Mean (SD); Median (IQR); n (%) | | | | |
| ^b^Wilcoxon rank sum test; t test; Pearson's chi-squared test; Fisher's exact test  *Indicates statistically significant difference (*p* <0.05)  **BMI**, body mass index; **CCI,** Charlson comorbidity index; **Malnutrition**, diagnosis by the Global Leadership Initiative on Malnutrition criteria; **FEV1/FVC,** forced expiratory volume in 1 second / forced vital capacity; **CS-30,** 30-second chair-stand test**; 6MWT,** 6-minute walk test**; HGS**, hand grip strength; **PPCs**, Postoperative pulmonary complications = pneumonia or sputum excretion difficulty; **LOS**, length of stay | | | | |
