# Supplementary material for: Preoperative exercise capacity as a predictor of postoperative pulmonary complications in patients with esophageal cancer: a multicenter observational cohort study
Source: Esophagus. 2026 Jun 30;23(4):791–800. doi: 10.1007/s10388-026-01226-y (PMC13723555; doi:10.1007/s10388-026-01226-y)
Supplement: Supplementary file 2 [file 10388_2026_1226_MOESM2_ESM.docx]

**Title: Preoperative exercise capacity as a predictor of postoperative respiratory complications in patients with esophageal cancer: a multicenter observational cohort study**

Journal name: Esophagus

Authors: Tomohiro Ikeda, MSc^1, 2^, Kazuhiro Noma, MD, PhD^3, 4^**^*^**, Kazuki Okura, PhD^5^, Hiroki Sato, PhD^6^, Sho Katayama^1^ ,Yusuke Takahashi, PhD^5^, Naoaki Maeda, MD, PhD^3, 4^, Shunsuke Tanabe, MD, PhD^3, 4^, Yusuke Sato, MD, PhD^7^, Yoshinori Fujiwara, MD, PhD^8^, Masanori Hamada, MD, PhD^1^, Toshifumi Ozaki, MD, PhD^1^, Tomohiko Nishigami, PhD^9^, Toshiyoshi Fujiwara, MD, PhD^3,4^

Affiliations: ^1^Department of Rehabilitation Medicine, Okayama University Hospital, 2-5-1 Shikatacho, Kita-ku, Okayama 700-8558, Japan

^2^Graduate School of Comprehensive Scientific Research, Prefectural University of Hiroshima, Gakuencho 1-1, Mihara, Hiroshima 723-0053, Japan

^3^Department of Gastroenterological Surgery, Graduate School of Medicine, Dentistry and Pharmaceutical Sciences, Okayama University, 2-5-1 Shikatacho, Kita-ku, Okayama 700-8558, Japan

^4^Center for Esophageal Disease, Okayama University Hospital, 2-5-1 Shikatacho, Kita-ku, Okayama 700-8558, Japan

^5^Department of Rehabilitation Medicine, Akita University Hospital, 44-2 Hasunuma, Hiroomote, Akita 010-8543, Japan

^6^Department of Physical Therapy, Faculty of Rehabilitation, Kawasaki University of Medical Welfare, Kurashiki, 288 Matsushima, Okayama 701-0193, Japan.

^7^Department of Esophageal Surgery, Akita University Hospital, 44-2 Hasunuma, Hiroomote, Akita 010-8543, Japan

^8^Department of Digestive Surgery, Kawasaki Medical School, Kurashiki, 577 Matsushima, Okayama 701-0192, Japan

^9^Department of Physical Therapy, Faculty of Health and Welfare, Prefectural University of Hiroshima, Gakuencho 1-1, Mihara, Hiroshima 723-0053, Japan

**^*^Corresponding author:** Kazuhiro Noma

Department of Gastroenterological Surgery, Graduate School of Medicine, Dentistry and Pharmaceutical Sciences, Okayama University, 2-5-1 Shikatacho, Kita-ku, Okayama 700-8558, Japan

E-mail address: [knoma@md.okayama-u.ac.jp](mailto:knoma@md.okayama-u.ac.jp)

Telephone number: +81-86-235-7752; Fax number: +81-86-235-7751

**Supplementary statistical code** **contents**

Page 3: Preliminary information

Page 3: Supplementary Statistical Code 1. McNemar’s test for paired comparison of missing data rates in CS-30 and 6MWT

Page 5: Supplementary Statistical Code 2. NRI and IDI analysis for the added predictive value of CS-30, 6MWT, HGS (hand grip strength), gait speed

Page 10: Supplementary Statistical Code 3. AUC estimation, likelihood ratio test, and ROC curve analysis

Page 17: Supplementary Statistical Code 4. ROC analysis with multiple imputation for cutoff determination

Page 21: Supplementary Statistical Code 5. Comparison of surgical and postoperative information according to CS-30 or 6MWT scores

Page 25: Supplementary Statistical Code 6. Comparison of clinical characteristics according to missing data status in the CS-30 and 6MWT

**Preliminary information**

\# All analyses were performed using R software (version 4.5.0)

\# Dataset name: df

\# Variables: WT6M, 6-minute walk distance; CS30, 30-second chair stand test; CCI, Charlson comorbidity index; cStage, clinical Stage; Malnutrition, diagnosis by the Global Leadership Initiative on Malnutrition criteria; FEV1/FVC, forced expiratory volume in 1 second / forced vital capacity

\# R packages used in addition to the standard ones are as follows: “missRanger”; “pROC”; “PredictABEL”, “mice”, “dplyr”, “gtsummary”

**Supplementary Statistical Code 1. McNemar’s test for paired comparison of missing data rates in CS-30 and 6MWT**

\# ------------------------------------------------------------

\# Step 1. Create a 2 × 2 contingency table of missingness

\# ------------------------------------------------------------

tab <- table(

is.na(df$WT6M),

is.na(df$CS30)

)

dimnames(tab) <- list(

`WT6M missing` = c("No", "Yes"),

`CS30 missing` = c("No", "Yes")

)

\# ------------------------------------------------------------

\# Step 2. Perform McNemar's test

\# Continuity correction was applied

\# ------------------------------------------------------------

mcnemar_res <- mcnemar.test(tab, correct = TRUE)

\# ------------------------------------------------------------

\# Step 3. Display the contingency table and test result

\# ------------------------------------------------------------

print(mcnemar_res)

**Supplementary Statistical Code 2. NRI and IDI analysis for the added predictive value of CS-30, 6MWT, HGS (hand grip strength), gait speed**

\# Outcome name: PPCs

\# Base model: Age + Sex + CCI + cStage + Malnutrition + FEV1FVC

\# Extended models:

\#	- Base model + CS30

\#	- Base model + WT6M

\#	- Base model + HGS

\#	- Base model + Gait speed

library(missRanger)

library(PredictABEL)

\# ------------------------------------------------------------

\# Step 1. Random forest imputation for missing values

\# ------------------------------------------------------------

set.seed(123)

df_imp <- missRanger(

df,

pmm.k = 3,

num.trees = 200,

verbose = TRUE

)

\# ------------------------------------------------------------

\# Step 2. Baseline model using established risk factors

\# ------------------------------------------------------------

GLM.1 <- glm(

PPCs ~ Age + Sex + CCI + cStage + Malnutrition + FEV1FVC,

data = df_imp,

family = binomial

)

\# ------------------------------------------------------------

\# Step 3. Reclassification analysis for the model adding CS30

\# ------------------------------------------------------------

GLM.2 <- glm(

PPCs ~ CS30 + Age + Sex + CCI + cStage + Malnutrition + FEV1FVC,

data = df_imp,

family = binomial

)

used_data_CS30 <- model.frame(GLM.2)

predRisk1_CS30 <- predict(GLM.1, newdata = used_data_CS30, type = "response")

predRisk2_CS30 <- predict(GLM.2, newdata = used_data_CS30, type = "response")

used_data_CS30$PPCs <- ifelse(used_data_CS30$PPCs %in% c(1, "1", TRUE), 1, 0)

value_CS30 <- as.vector(quantile(predRisk1_CS30, na.rm = TRUE))

cutoff_CS30 <- c(0, value_CS30[2], value_CS30[3], value_CS30[4], 1)

result_CS30 <- reclassification(

data	= used_data_CS30,

cOutcome = which(names(used_data_CS30) == "PPCs"),

predrisk1 = predRisk1_CS30,

predrisk2 = predRisk2_CS30,

cutoff	= cutoff_CS30

)

print(result_CS30)

\# ------------------------------------------------------------

\# Step 4. Reclassification analysis for the model adding WT6M

\# ------------------------------------------------------------

GLM.3 <- glm(

PPCs ~ WT6M + Age + Sex + CCI + cStage + Malnutrition + FEV1FVC,

data = df_imp,

family = binomial

)

used_data_WT6M <- model.frame(GLM.3)

predRisk1_WT6M <- predict(GLM.1, newdata = used_data_WT6M, type = "response")

predRisk2_WT6M <- predict(GLM.3, newdata = used_data_WT6M, type = "response")

used_data_WT6M$PPCs <- ifelse(used_data_WT6M$PPCs %in% c(1, "1", TRUE), 1, 0)

value_WT6M <- as.vector(quantile(predRisk1_WT6M, na.rm = TRUE))

cutoff_WT6M <- c(0, value_WT6M[2], value_WT6M[3], value_WT6M[4], 1)

result_WT6M <- reclassification(

data	= used_data_WT6M,

cOutcome = which(names(used_data_WT6M) == "PPCs"),

predrisk1 = predRisk1_WT6M,

predrisk2 = predRisk2_WT6M,

cutoff	= cutoff_WT6M

)

print(result_WT6M)

\# ------------------------------------------------------------

\# Step 5. Reclassification analysis for the model adding HGS

\# ------------------------------------------------------------

GLM.4 <- glm(

PPCs ~ HGS + Age + Sex + CCI + cStage + Malnutrition + FEV1FVC,

data = df_imp,

family = binomial

)

used_data_HGS <- model.frame(GLM.4)

predRisk1_HGS <- predict(GLM.1, newdata = used_data_HGS, type = "response")

predRisk2_HGS <- predict(GLM.4, newdata = used_data_HGS, type = "response")

used_data_HGS$PPCs <- ifelse(used_data_HGS$PPCs %in% c(1, "1", TRUE), 1, 0)

value_HGS <- as.vector(quantile(predRisk1_HGS, na.rm = TRUE))

cutoff_HGS <- c(0, value_HGS[2], value_HGS[3], value_HGS[4], 1)

result_HGS <- reclassification(

data	= used_data_HGS,

cOutcome = which(names(used_data_HGS) == "PPCs"),

predrisk1 = predRisk1_HGS,

predrisk2 = predRisk2_HGS,

cutoff	= cutoff_HGS

)

print(result_HGS)

\# ------------------------------------------------------------

\# Step 6. Reclassification analysis for the model adding Gait speed

\# ------------------------------------------------------------

GLM.5 <- glm(

PPCs ~ Gait speed + Age + Sex + CCI + cStage + Malnutrition + FEV1FVC,

data = df_imp,

family = binomial

)

used_data_GS <- model.frame(GLM.5)

predRisk1_GS <- predict(GLM.1, newdata = used_data_GS, type = "response")

predRisk2_GS <- predict(GLM.5, newdata = used_data_GS, type = "response")

used_data_GS$PPCs <- ifelse(used_data_GS$PPCs %in% c(1, "1", TRUE), 1, 0)

value_GS <- as.vector(quantile(predRisk1_GS, na.rm = TRUE))

cutoff_GS <- c(0, value_GS[2], value_GS[3], value_GS[4], 1)

result_GS <- reclassification(

data	= used_data_GS,

cOutcome = which(names(used_data_GS) == "PPCs"),

predrisk1 = predRisk1_GS,

predrisk2 = predRisk2_GS,

cutoff	= cutoff_GS

)

print(result_GS)

**Supplementary Statistical Code 3. AUC estimation, likelihood ratio test, and ROC curve analysis**

\# AUC estimation, likelihood ratio test, and ROC curve analysis for evaluating the added predictive value of CS-30 and 6MWT

\# Dataset name: df

\# Outcome name: PPCs

\# Base model: Age + Sex + CCI + cStage + Malnutrition + FEV1FVC

\# Extended models:

\#	- Base model + CS30

\#	- Base model + WT6M

\#	- Base model + HGS

\#	- Base model + Gait speed

\# ------------------------------------------------------------

\# Step 1. Random forest imputation for missing values

\# ------------------------------------------------------------

set.seed(123)

df_imp <- missRanger(

df,

pmm.k = 3,

num.trees = 100,

verbose = 1

)

\# ------------------------------------------------------------

\# Step 2. Fit logistic regression models

\# ------------------------------------------------------------

\# Baseline model using established risk factors

fit0 <- glm(

PPCs ~ Age + Sex + CCI + cStage + Malnutrition + FEV1FVC,

data = df_imp,

family = binomial

)

\# Extended model adding CS30

fit1 <- glm(

PPCs ~ CS30 + Age + Sex + CCI + cStage + Malnutrition + FEV1FVC,

data = df_imp,

family = binomial

)

\# Extended model adding WT6M

fit2 <- glm(

PPCs ~ WT6M + Age + Sex + CCI + cStage + Malnutrition + FEV1FVC,

data = df_imp,

family = binomial

)

\# Extended model adding HGS

fit3 <- glm(

PPCs ~ HGS + Age + Sex + CCI + cStage + Malnutrition + FEV1FVC,

data = df_imp,

family = binomial

)

\# Extended model adding Gait speed

fit4 <- glm(

PPCs ~ Gait speed + Age + Sex + CCI + cStage + Malnutrition + FEV1FVC,

data = df_imp,

family = binomial

)

\# ------------------------------------------------------------

\# Step 3. Likelihood ratio tests

\# ------------------------------------------------------------

anova(fit0, fit1, test = "Chisq")

anova(fit0, fit2, test = "Chisq")

anova(fit0, fit3, test = "Chisq")

anova(fit0, fit4, test = "Chisq")

\# ------------------------------------------------------------

\# Step 4. ROC curve analysis and AUC estimation

\# ------------------------------------------------------------

roc0 <- roc(df_imp$PPCs, predict(fit0, type = "response"))

roc1 <- roc(df_imp$PPCs, predict(fit1, type = "response"))

roc2 <- roc(df_imp$PPCs, predict(fit2, type = "response"))

roc3 <- roc(df_imp$PPCs, predict(fit3, type = "response"))

roc4 <- roc(df_imp$PPCs, predict(fit4, type = "response"))

auc0 <- as.numeric(auc(roc0))

auc1 <- as.numeric(auc(roc1))

auc2 <- as.numeric(auc(roc2))

auc3 <- as.numeric(auc(roc3))

auc4 <- as.numeric(auc(roc4))

\# Optional: 95% confidence intervals for AUC

ci.auc(roc0)

ci.auc(roc1)

ci.auc(roc2)

ci.auc(roc3)

ci.auc(roc4)

\# Optional: DeLong tests for AUC comparison

roc.test(roc0, roc1, method = "delong")

roc.test(roc0, roc2, method = "delong")

roc.test(roc0, roc3, method = "delong")

roc.test(roc0, roc4, method = "delong")

\# ------------------------------------------------------------

\# Step 5. Extract ROC coordinates

\# ------------------------------------------------------------

c0 <- coords(roc0, x = "all", ret = c("specificity", "sensitivity"), transpose = FALSE)

c1 <- coords(roc1, x = "all", ret = c("specificity", "sensitivity"), transpose = FALSE)

c2 <- coords(roc2, x = "all", ret = c("specificity", "sensitivity"), transpose = FALSE)

c3 <- coords(roc3, x = "all", ret = c("specificity", "sensitivity"), transpose = FALSE)

c4 <- coords(roc4, x = "all", ret = c("specificity", "sensitivity"), transpose = FALSE)

fpr0 <- 1 - c0$specificity

tpr0 <- c0$sensitivity

fpr1 <- 1 - c1$specificity

tpr1 <- c1$sensitivity

fpr2 <- 1 - c2$specificity

tpr2 <- c2$sensitivity

fpr3 <- 1 - c3$specificity

tpr3 <- c3$sensitivity

fpr4 <- 1 - c4$specificity

tpr4 <- c4$sensitivity

\# ------------------------------------------------------------

\# Step 6. Plot ROC curves

\# ------------------------------------------------------------

par(mar = c(4.5, 4.5, 1.2, 1.2))

plot(

fpr0, tpr0,

type = "l",

lty = 1,

lwd = 2.5,

col = "black",

xlab = "1 - Specificity",

ylab = "Sensitivity",

xlim = c(0, 1),

ylim = c(0, 1),

xaxs = "i",

yaxs = "i"

)

lines(fpr1, tpr1, lty = 1, lwd = 1.5, col = "red")

lines(fpr2, tpr2, lty = 4, lwd = 2.5, col = "blue")

lines(fpr3, tpr3, lty = 2, lwd = 2.0, col = "darkgreen")

lines(fpr4, tpr4, lty = 5, lwd = 2.0, col = "purple")

abline(0, 1, lty = 3, col = "gray70")

\# Add point markers to the ROC curve of the CS30 model

idx <- unique(round(seq(1, length(fpr1), length.out = 120)))

points(fpr1[idx], tpr1[idx], pch = 16, cex = 0.55, col = "red")

legend(

"bottomright",

legend = c(

sprintf("Baseline model (AUC = %.3f)", auc0),

sprintf("Baseline model + CS30 (AUC = %.3f)", auc1),

sprintf("Baseline model + WT6M (AUC = %.3f)", auc2),

sprintf("Baseline model + HGS (AUC = %.3f)", auc3),

sprintf("Baseline model + Gait speed (AUC = %.3f)", auc4)

),

lty = c(1, 1, 4, 2, 5),

lwd = c(2.5, 1.5, 2.5, 2.0, 2.0),

col = c("black", "red", "blue", "darkgreen", "purple"),

pch = c(NA, 16, NA, NA, NA),

pt.cex = 0.8,

bty = "n",

seg.len = 3

)

**Supplementary Statistical Code 4. ROC analysis with multiple imputation for cutoff determination**

\# Dataset name: df

\# Outcome name: PPCs

\# Names of predictor variables: CS30, WT6M

library(mice)

library(pROC)

set.seed(123)

\# ------------------------------------------------------------

\# Step 1. Multiple imputation

\# ------------------------------------------------------------

imp <- mice(

df,

m = 20,

method = "pmm",

maxit = 20,

seed = 123,

printFlag = FALSE

)

\# ------------------------------------------------------------

\# Step 2. ROC analysis in each imputed dataset

\# ------------------------------------------------------------

auc_cs30 <- numeric(20)

auc_wt6m <- numeric(20)

cut_cs30 <- numeric(20)

cut_wt6m <- numeric(20)

sens_cs30 <- numeric(20)

spec_cs30 <- numeric(20)

sens_wt6m <- numeric(20)

spec_wt6m <- numeric(20)

for (i in 1:20) {

dat_i <- complete(imp, i)

\# ROC for CS30

roc_cs30 <- roc(dat_i$PPCs, dat_i$CS30, quiet = TRUE)

auc_cs30[i] <- as.numeric(auc(roc_cs30))

best_cs30 <- coords(

roc_cs30,

x = "best",

best.method = "youden",

ret = c("threshold", "sensitivity", "specificity"),

transpose = FALSE

)

cut_cs30[i] <- best_cs30$threshold

sens_cs30[i] <- best_cs30$sensitivity

spec_cs30[i] <- best_cs30$specificity

\# ROC for WT6M

roc_wt6m <- roc(dat_i$PPCs, dat_i$WT6M, quiet = TRUE)

auc_wt6m[i] <- as.numeric(auc(roc_wt6m))

best_wt6m <- coords(

roc_wt6m,

x = "best",

best.method = "youden",

ret = c("threshold", "sensitivity", "specificity"),

transpose = FALSE

)

cut_wt6m[i] <- best_wt6m$threshold

sens_wt6m[i] <- best_wt6m$sensitivity

spec_wt6m[i] <- best_wt6m$specificity

}

\# ------------------------------------------------------------

\# Step 3. Summarize results across imputations

\# ------------------------------------------------------------

roc_summary <- data.frame(

Predictor = c("CS30", "WT6M"),

Mean_AUC = c(mean(auc_cs30), mean(auc_wt6m)),

SD_AUC = c(sd(auc_cs30), sd(auc_wt6m)),

Mean_cutoff = c(mean(cut_cs30), mean(cut_wt6m)),

SD_cutoff = c(sd(cut_cs30), sd(cut_wt6m)),

Mean_sensitivity = c(mean(sens_cs30), mean(sens_wt6m)),

Mean_specificity = c(mean(spec_cs30), mean(spec_wt6m))

)

print(roc_summary)

**Supplementary Statistical Code 5. Comparison of surgical and postoperative information according to CS-30 or 6MWT scores**

\# Dataset name: df

library(dplyr)

library(gtsummary)

\# ------------------------------------------------------------

\# 1. Create grouping variables

\# ------------------------------------------------------------

df_tab <- df_imp %>%

mutate(

CS30_group = ifelse(CS30 < 15, "<15", "≥15"),

WT6M_group = ifelse(WT6M < 510, "<510", "≥510")

)

\# 2. Specify variables

cat_vars <- c(

"LND (3 area)",

"Robot",

"Thoracoscopic",

"Thoracotomy",

"Laparoscopic",

"Laparotomy",

"Goal",

"Pneumonia",

"Pneumonia/Sputum excretion difficulty",

"Anastomotic Leakage",

"RLNP",

"SSI",

"Poaf"

)

cont_vars <- c(

"Time (min)",

"Bleeding (ml)",

"First gait (days)",

"LOS (days)"

)

vars <- c(cat_vars, cont_vars)

\# ------------------------------------------------------------

\# 3. Check normality of continuous variables in the overall sample

\# ------------------------------------------------------------

normality_results <- lapply(cont_vars, function(v) {

x <- df_tab[[v]]

x <- x[!is.na(x)]

data.frame(

Variable = v,

Shapiro_Wilk_p = if (length(x) >= 3) shapiro.test(x)$p.value else NA

)

}) %>%

bind_rows()

normality_results

\# ------------------------------------------------------------

\# 4. Table 3. Compare groups based on the CS30 cutoff

\# ------------------------------------------------------------

Table 3 <-

df_tab %>%

select(CS30_group, all_of(vars)) %>%

tbl_summary(

by = CS30_group,

type = all_of(cat_vars) ~ "categorical",

statistic = list(

all_of(cont_vars) ~ "{median} ({p25}, {p75})",

all_categorical() ~ "{n} ({p}%)"

),

missing = "no"

) %>%

add_p(

test = list(

all_of(cont_vars) ~ "wilcox.test",

all_of(cat_vars) ~ "fisher.test"

)

) %>%

Table 3

\# ------------------------------------------------------------

\# 5. Table 4. Compare groups based on the WT6M cutoff

\# ------------------------------------------------------------

Table 4 <-

df_tab %>%

select(WT6M_group, all_of(vars)) %>%

tbl_summary(

by = WT6M_group,

type = all_of(cat_vars) ~ "categorical",

statistic = list(

all_of(cont_vars) ~ "{median} ({p25}, {p75})",

all_categorical() ~ "{n} ({p}%)"

),

missing = "no"

) %>%

add_p() %>%

Table 4

**Supplementary Statistical Code 6.** **Comparison of clinical characteristics according to missing data status in the CS-30 and 6MWT**

\# Dataset name: df

library(dplyr)

library(gtsummary)

\# ------------------------------------------------------------

\# 1. Create missingness groups

\# ------------------------------------------------------------

df_tab <- df %>%

mutate(

CS30_missing = ifelse(is.na(CS30), "Missing", "Not missing"),

WT6M_missing = ifelse(is.na(WT6M), "Missing", "Not missing")

)

\# ------------------------------------------------------------

\# 2. Specify variables for comparison

\# ------------------------------------------------------------

cat_vars <- c(

"Sex",

"Location",

"Histology",

"Brinkman index（≥800）",

"CCI",

"cStage",

"Neoadjuvant therapy",

"Malnutrition",

"PPCs",

"Pneumonia"

)

cont_vars <- c(

"Age",

"BMI",

"Hemoglobin",

"Albumin",

"CS30",

"WT6M",

"HGS",

"Gait speed",

"FEV1FVC"

)

vars <- c(cat_vars, cont_vars)

vars <- vars[vars %in% names(df_tab)]

cat_vars <- cat_vars[cat_vars %in% names(df_tab)]

cont_vars <- cont_vars[cont_vars %in% names(df_tab)]

\# ------------------------------------------------------------

\# 3. Function to select test for continuous variables

\#	If both groups are approximately normal -> t.test

\#	Otherwise -> wilcox.test

\# ------------------------------------------------------------

select_cont_test <- function(data, group_var, cont_vars) {

test_list <- list()

for (v in cont_vars) {

dat_sub <- data %>%

select(all_of(group_var), all_of(v)) %>%

filter(!is.na(.data[[group_var]]), !is.na(.data[[v]]))

group_levels <- unique(dat_sub[[group_var]])

if (length(group_levels) != 2) {

test_list[[v]] <- "wilcox.test"

next

}

x1 <- dat_sub %>% filter(.data[[group_var]] == group_levels[1]) %>% pull(all_of(v))

x2 <- dat_sub %>% filter(.data[[group_var]] == group_levels[2]) %>% pull(all_of(v))

p1 <- if (length(x1) >= 3 && length(x1) <= 5000) shapiro.test(x1)$p.value else NA

p2 <- if (length(x2) >= 3 && length(x2) <= 5000) shapiro.test(x2)$p.value else NA

if (!is.na(p1) && !is.na(p2) && p1 >= 0.05 && p2 >= 0.05) {

test_list[[v]] <- "t.test"

} else {

test_list[[v]] <- "wilcox.test"

}

}

test_list

}

\# ------------------------------------------------------------

\# 4. Create test lists

\# ------------------------------------------------------------

cont_tests_cs30 <- select_cont_test(df_tab, "CS30_missing", cont_vars)

cont_tests_wt6m <- select_cont_test(df_tab, "WT6M_missing", cont_vars)

test_list_cs30 <- c(

cont_tests_cs30,

setNames(as.list(rep("fisher.test", length(cat_vars))), cat_vars)

)

test_list_wt6m <- c(

cont_tests_wt6m,

setNames(as.list(rep("fisher.test", length(cat_vars))), cat_vars)

)

\# ------------------------------------------------------------

\# 5. Optional: show which tests were selected

\# ------------------------------------------------------------

cont_tests_cs30

cont_tests_wt6m

\# ------------------------------------------------------------

\# 6. eTable 4. Comparison of clinical characteristics

\#	according to missing data status in the CS-30

\# ------------------------------------------------------------

etable4 <-

df_tab %>%

select(CS30_missing, all_of(vars)) %>%

tbl_summary(

by = CS30_missing,

type = all_of(cat_vars) ~ "categorical",

statistic = list(

all_of(cont_vars) ~ "{median} ({p25}, {p75})",

all_categorical() ~ "{n} ({p}%)"

),

missing = "no"

) %>%

add_p(test = test_list_cs30) %>%

modify_caption("**eTable 4. Missing data in the CS-30 and clinical characteristics**") %>%

etable4

\# ------------------------------------------------------------

\# 7. eTable 5. Comparison of clinical characteristics

\#	according to missing data status in the 6MWT

\# ------------------------------------------------------------

etable5 <-

df_tab %>%

select(WT6M_missing, all_of(vars)) %>%

tbl_summary(

by = WT6M_missing,

type = all_of(cat_vars) ~ "categorical",

statistic = list(

all_of(cont_vars) ~ "{median} ({p25}, {p75})",

all_categorical() ~ "{n} ({p}%)"

),

missing = "no"

) %>%

add_p(test = test_list_wt6m) %>%

modify_caption("**eTable 5. Missing data in the 6MWT and clinical characteristics**") %>%

etable5
