# Supplementary material for: Preoperative exercise capacity as a predictor of postoperative pulmonary complications in patients with esophageal cancer: a multicenter observational cohort study
Source: Esophagus. 2026 Jun 30;23(4):791–800. doi: 10.1007/s10388-026-01226-y (PMC13723555; doi:10.1007/s10388-026-01226-y)
Supplement: Supplementary file 3 [file 10388_2026_1226_MOESM3_ESM.docx]

**Title: Preoperative exercise capacity as a predictor of postoperative respiratory complications in patients with esophageal cancer: a multicenter observational cohort study**

Journal name: Esohagus

Authors: Tomohiro Ikeda, MSc^1, 2^, Kazuhiro Noma, MD, PhD^3, 4^**^*^**, Kazuki Okura, PhD^5^, Hiroki Sato, PhD^6^, Sho Katayama^1^ ,Yusuke Takahashi, PhD^5^, Naoaki Maeda, MD, PhD^3, 4^, Shunsuke Tanabe, MD, PhD^3, 4^, Yusuke Sato, MD, PhD^7^, Yoshinori Fujiwara, MD, PhD^8^, Masanori Hamada, MD, PhD^1^, Toshifumi Ozaki, MD, PhD^1^, Tomohiko Nishigami, PhD^9^, Toshiyoshi Fujiwara, MD, PhD^3,4^

Affiliations: ^1^Department of Rehabilitation Medicine, Okayama University Hospital, 2-5-1 Shikatacho, Kita-ku, Okayama 700-8558, Japan

^2^Graduate School of Comprehensive Scientific Research, Prefectural University of Hiroshima, Gakuencho 1-1, Mihara, Hiroshima 723-0053, Japan

^3^Department of Gastroenterological Surgery, Graduate School of Medicine, Dentistry and Pharmaceutical Sciences, Okayama University, 2-5-1 Shikatacho, Kita-ku, Okayama 700-8558, Japan

^4^Center for Esophageal Disease, Okayama University Hospital, 2-5-1 Shikatacho, Kita-ku, Okayama 700-8558, Japan

^5^Department of Rehabilitation Medicine, Akita University Hospital, 44-2 Hasunuma, Hiroomote, Akita 010-8543, Japan

^6^Department of Physical Therapy, Faculty of Rehabilitation, Kawasaki University of Medical Welfare, Kurashiki, 288 Matsushima, Okayama 701-0193, Japan.

^7^Department of Esophageal Surgery, Akita University Hospital, 44-2 Hasunuma, Hiroomote, Akita 010-8543, Japan

^8^Department of Digestive Surgery, Kawasaki Medical School, Kurashiki, 577 Matsushima, Okayama 701-0192, Japan

^9^Department of Physical Therapy, Faculty of Health and Welfare, Prefectural University of Hiroshima, Gakuencho 1-1, Mihara, Hiroshima 723-0053, Japan

**^*^Corresponding author:** Kazuhiro Noma

Department of Gastroenterological Surgery, Graduate School of Medicine, Dentistry and Pharmaceutical Sciences, Okayama University, 2-5-1 Shikatacho, Kita-ku, Okayama 700-8558, Japan

E-mail address: [knoma@md.okayama-u.ac.jp](mailto:knoma@md.okayama-u.ac.jp)

Telephone number: +81-86-235-7752; Fax number: +81-86-235-7751

eFig. 1. ROC curve for evaluating the postoperative pneumonia prediction model including the CS-30 and 6 MWT (random forest imputation analysis).

**ROC,** receiver operating characteristic; **Established model**, model including established risk factors for postoperative pneumonia: age, sex, Charlson Comorbidity Index ≥2, clinical stage ≥II, malnutrition, and forced vital capacity/forced expiratory volume in one second; **AUC**, area under the curve; **CS-30,** 30-second chair-stand test**; 6MWT,** 6-minute walk test

eFig. 2. ROC curve for evaluating the PPCs prediction model including the CS-30 and 6 MWT (complete case analysis).

**ROC,** receiver operating characteristic; **PPCs**, Postoperative pulmonary complications = pneumonia or sputum excretion difficulty; **Established model**, model including established risk factors for PPCs: age, sex, Charlson Comorbidity Index ≥2, clinical stage ≥II, malnutrition, and forced vital capacity/forced expiratory volume in one second; **AUC**, area under the curve; **CS-30,** 30-second chair-stand test**; 6MWT,** 6-minute walk test
